# Intense Pulsed Light Improves Facial Telangiectasia and Acne‐Induced Post‐Inflammatory Erythema in Systemic Lupus Erythematosus Patient: A Case Report

**DOI:** 10.1111/jocd.70389

**Published:** 2025-08-22

**Authors:** Zhen Li, Jiejie Lu, Miaoting Wu

**Affiliations:** ^1^ Plastic and Cosmetic Center The Fifth People's Hospital of Hainan Province Haikou Hainan Province China

**Keywords:** acne vulgaris, intense pulsed light therapy, post‐inflammatory erythema, systemic lupus erythematosus, telangiectasia

## Abstract

**Background:**

Systemic lupus erythematosus (SLE) is a chronic, multisystem autoimmune disease. Standard treatment typically involves corticosteroids, antimalarials, and immunosuppressants; however, long‐term corticosteroid use can lead to adverse cutaneous effects, including facial telangiectasia and acne vulgaris. While medical management often addresses active acne, residual telangiectasia and post‐inflammatory erythema (PIE) frequently persist as challenging cosmetic concerns.

**Aim:**

To study the efficacy of intense pulsed light (IPL) in treating facial telangiectasia and PIE in patients with SLE; to record the possible adverse reactions of this treatment.

**Methods:**

We present a case of a 21‐year‐old female SLE patient with facial telangiectasia and PIE. The treatment protocol incorporated five sessions of IPL therapy using dual‐band vascular filters (530–650 nm and 900–1200 nm) along with a 590 nm filter.

**Results:**

After five sessions of IPL treatment, the facial telangiectasia and PIE of the patient were improved. During the 6‐month follow‐up period, no adverse reactions and worsening of SLE were observed.

**Conclusions:**

Low‐energy IPL may be a safe and effective therapeutic option for managing steroid‐induced telangiectasia and acne‐related PIE in SLE patients.

## 1 Introduction

Systemic lupus erythematosus (SLE) is a chronic, multisystem autoimmune disorder characterized by a relapsing–remitting course [1]. Standard treatment involves corticosteroids, antimalarials, and immunosuppressants [2]. However, prolonged corticosteroid use is associated with adverse cutaneous effects, including acne vulgaris, facial erythema, and telangiectasia [3, 4]. While acne vulgaris is a common inflammatory disease [5], it triggers excessive melanogenesis and abnormal melanin deposition, resulting in post‐inflammatory erythema (PIE) [6]. Conventional topical and oral therapies often prove ineffective for telangiectasia and acne‐induced PIE, necessitating alternative interventions such as photoelectric therapy.

Herein, we present a 21‐year‐old female SLE patient who achieved improvement in facial telangiectasia and acne‐induced PIE following five sessions of low‐energy intense pulsed light (IPL) therapy utilizing dual‐band vascular filters (530–650 nm and 900–1200 nm) and a 590 nm filter.

## 2 Case Report

**FIGURE 1:**
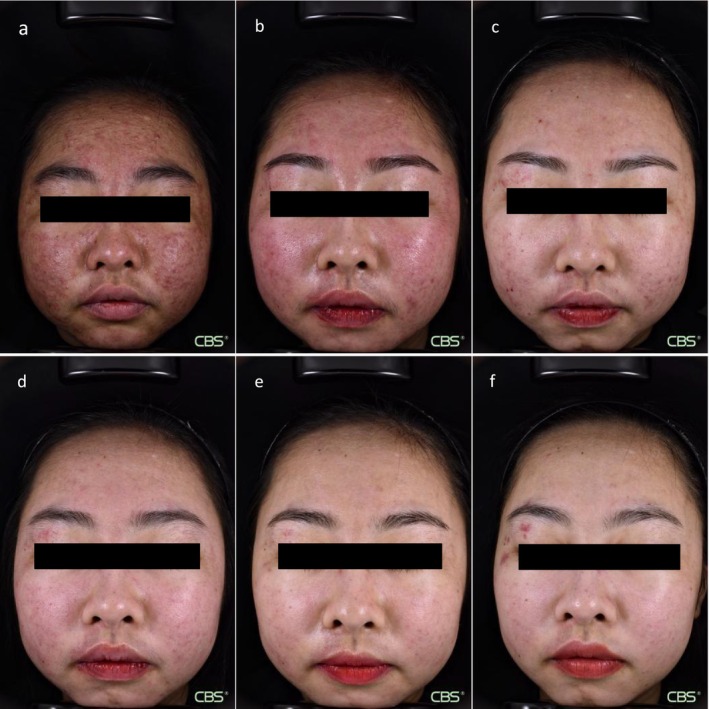
Clinical photographs show the progression of the patient's condition. (a) Premedication (CEA 4 points); (b) Before IPL treatment; (c) After IPL treatment 4; (d) 8; (e) 12; (f) 16 weeks (CEA 2 points).

A 21‐year‐old female patient with a 1‐year history of recurrent severe inflammatory acne vulgaris (Pillsbury grade IV), characterized by facial erythematous papules, pustules, and nodular lesions (Figure 1a), presented to the dermatology department in March 2024. The patient had been diagnosed with SLE 10 years prior and had received long‐term maintenance therapy with oral low‐dose methylprednisolone, hydroxychloroquine, and other medications. She also reported facial telangiectasia persisting for 8 years. The patient was administered oral isotretinoin soft capsules (10 mg twice daily) for acne vulgaris. After 6 weeks of therapy, facial papules, pustules, and nodules demonstrated marked improvement (Figure 1b); however, facial telangiectasia and acne‐induced PIE exacerbated the patient's anxiety.

**FIGURE 2:**
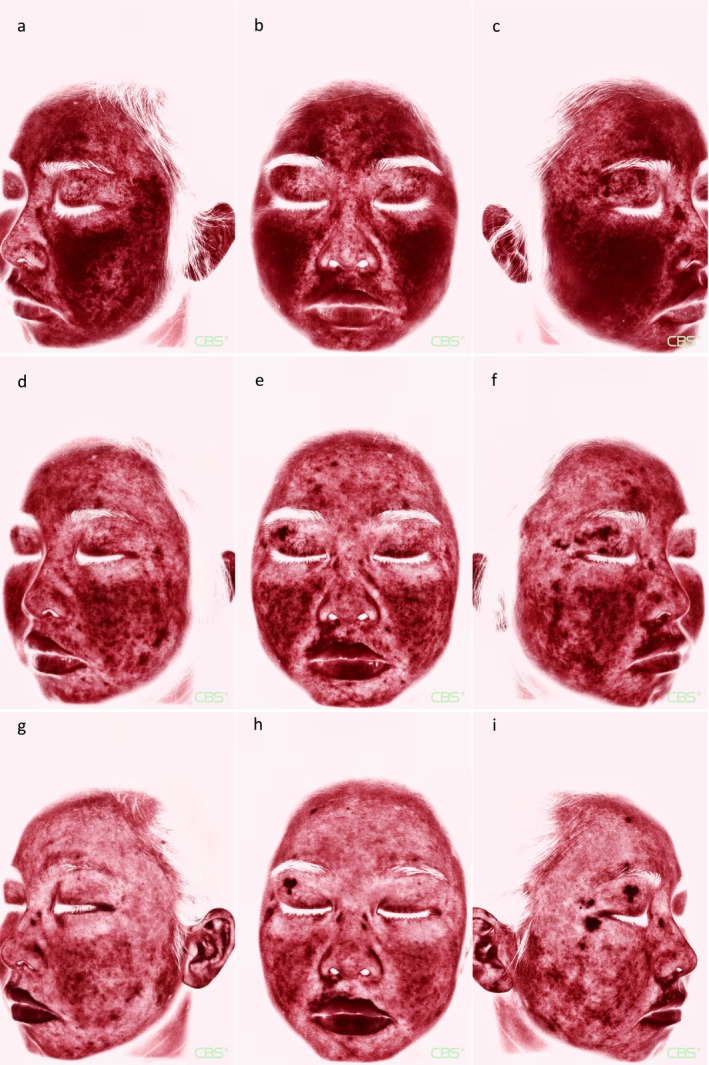
Images during IPL treatment (CBS). (a‑c) Before IPL treatment; (d‑f) After IPL treatment 8 weeks; (g‑i) After IPL treatment 16 weeks.

Therefore, we decided to use IPL (Lumenis M22 System, Lumenis Ltd.) to perform facial treatment on the patient. The parameter settings were as follows: [1] M22 590 filter (590–1200 nm): three pulses, pulse width 4–5 ms, pulse delay 40–50 ms, energy density 10–12 J/cm [2], and spot size 15 × 35 mm. [2] M22 dual‐band vascular filters (530–650 nm, 900–1200 nm): three pulses, pulse width 4–5 ms, pulse delay 40–50 ms, energy density 10–12 J/cm^2^, and spot size 15 × 35 mm. Cooling was maintained at 2°C–5°C, with one pass per filter and 10%–20% overlap. Sun protection was advised for 2 weeks before and post‐procedure. Following five monthly treatment sessions, according to the Clinical erythema assessment (CEA) rating scale, the patient's severe erythema (CEA 4 points) improved to mild erythema (CEA 2 points), indicating an improvement (Figures 1c–f and 2). Transient mild erythema occurred during treatment, but no severe adverse effects were observed.

During the six‐month follow‐up period, the patient had facial eruptions without recurrence. Comprehensive laboratory monitoring, encompassing complete blood count, renal function panels, complement levels, and anti‐dsDNA antibody titers, was performed throughout the therapeutic course and follow‐up. Clinical assessment incorporating both laboratory parameters and symptomatology revealed a Systemic Lupus Erythematosus Disease Activity Index (SLEDAI) score persistently below 4 points, confirming sustained disease stability.

## 3 Discussion

SLE is a chronic, multisystem autoimmune disease characterized by a relapsing–remitting course. It predominantly affects women of childbearing age, with a female‐to‐male ratio of 9:1 [1]. The exact etiology of SLE remains incompletely understood; however, photosensitivity is a hallmark feature of the disease. Ultraviolet (UV) light exposure and inflammatory responses are recognized as primary etiological factors for photosensitivity [7]. Standard therapeutic regimens encompass topical corticosteroids and antimalarials, while systemic therapies include thalidomide, auranofin, retinoids, dapsone, and immunosuppressive agents [2]. Nevertheless, various adverse cutaneous reactions, including acne vulgaris and facial erythem, have been documented during corticosteroid therapy [3]. Ghouse et al. reported that topical facial corticosteroid use over 6 months was associated with corticosteroid‐induced acne (83.6%), followed by facial erythema (50.9%) and telangiectasia (47.8%) [4]. Consequently, we posit that the acne and telangiectasia observed in this SLE patient are likely attributable to prolonged corticosteroid treatment.

Acne vulgaris is a chronic inflammatory skin disease affecting the pilosebaceous units, predominantly adolescents, with an estimated global prevalence of 9.4% [5]. PIE is one of the most common acne‐related complications, characterized by telangiectasia and erythematous papules that typically persist after inflammatory acne resolution. These vascular lesions are superficially located and exhibit an erythematous appearance attributed to superficial vasculature [6]. Although facial PIE may gradually improve over time, complete resolution is often unattainable in certain cases [8]. Conventional therapies, such as chemical peels and isotretinoin, are effective for PIE; however, their prolonged duration and adverse effects frequently limit tolerability [9]. Consequently, when the presented SLE patient developed concurrent telangiectasia and acne‐induced PIE, we pursued alternative therapeutic strategies. Energy‐based devices are increasingly favored over traditional pharmacotherapy due to their efficacy, minimal downtime, and reduced adverse effects.

Laser therapies have demonstrated efficacy across various manifestations of cutaneous lupus erythematosus (CLE), particularly in discoid lupus erythematosus (DLE) cases [10, 11, 12]. In a retrospective analysis by Eckbæk et al., 16 DLE patients underwent treatment with pulsed dye laser (PDL) and low‐intensity IPL therapy, with 14 patients exhibiting significant improvement in pruritus, erythema, desquamation, scarring, and pain [13]. Levy documented a case involving a 33‐year‐old female SLE patient presenting with chronic facial eruptions and rosacea symptoms. Following two sessions of IPL therapy, the patient achieved 75% lesion resolution without any reported adverse effects [14].

IPL utilizes a noncoherent, polychromatic broad‐spectrum flashlamp emitting wavelengths between approximately 400 and 1,200 nm. By employing specific filters, IPL can selectively target chromophores, enabling its application in treating vascular and pigmentary skin disorders, photoaging, and appendage‐related conditions [15]. In clinical settings, the condition of SLE is related to ultraviolet radiation. However, high‐energy‐density visible light may also have an impact. When using visible light emitted by IPL, it may lead to aggravation of SLE skin lesions. Therefore, caution should be exercised when using strong pulsed light therapy.

In our report, we opted for a cautious approach using the M22 system's 590‐nm filter with low fluence (10–12 J/cm^2^) and vascular filters to treat telangiectasia and acne‐induced PIE. The M22 590‐nm filter emits broad‐spectrum IPL with a longer pulse width, reduced fluence, and larger spot size compared to conventional lasers; thereby minimizing downtime and adverse effects [16]. The M22 vascular filter operates as a dual narrow‐band IPL system, emitting wavelengths of 530–650 nm (short‐wave band) and 900–1200 nm (long‐wave band). The short‐wave band maximizes absorption by deoxyhemoglobin and oxyhemoglobin while minimizing melanin interference, whereas the long‐wave band targets oxygenated hemoglobin's secondary absorption peak at deeper tissue layers. By excluding the 650–900 nm range, this filter enhances chromophore‐specific energy delivery [17, 18]. Following five monthly treatment sessions, the patient experienced improvements in erythema and capillary dilatation. It is worth noting that during the low‐fluence IPL therapy and in the follow‐up examination conducted 6 months later, the patient's SLE condition remained stable.

## 4 Conclusion

This represents the first documented application of a low‐energy IPL system for treating facial telangiectasia and acne‐induced PIE in an SLE patient. However, as this is a single case report, this study has certain limitations. Meanwhile, treatment administration necessitates continuous monitoring of SLE disease activity coupled with personalized clinical evaluation.

## Author Contributions

**Zhen Li:** manuscript drafting and editing, statistical analysis. **Jiejie Lu:** patient treatment, data collection, and manuscript review. **Miaoting Wu:** data collection, analysis, and interpretation. All authors reviewed and approved the final manuscript.

## Ethics Statement

This study was conducted under the principles outlined in the Declaration of Helsinki. All procedures performed were in compliance with ethical standards and guidelines for medical research involving human subjects. Written informed consent was obtained from the patient for participation in the study and for publication of the case details and accompanying images, with explicit permission granted for image reuse.

## Consent

The patient had given written informed consent for the publication of her clinical details and accompanying images.

## Conflicts of Interest

The authors declare no conflicts of interest.

## Data Availability

The authors have nothing to report.
